# Characterization of *Clostridium difficile* isolates using capillary gel electrophoresis-based PCR ribotyping

**DOI:** 10.1099/jmm.0.47714-0

**Published:** 2008-11

**Authors:** A. Indra, S. Huhulescu, M. Schneeweis, P. Hasenberger, S. Kernbichler, A. Fiedler, G. Wewalka, F. Allerberger, E. J. Kuijper

**Affiliations:** 1 Austrian Agency for Health and Food Safety (AGES), Vienna, Austria; 2 EDV-Tüftler, Vienna, Austria; 3 Department of Medical Microbiology, Center of Infectious Diseases, Leiden University Medical Center, Leiden, The Netherlands

## Abstract

We have developed a *Clostridium difficile* PCR ribotyping method based on capillary gel electrophoresis and have compared it with conventional PCR ribotyping. A total of 146 *C. difficile* isolates were studied: five isolates were reference strains (PCR ribotypes 001, 014, 017, 027 and 053); 141 were clinical isolates comprising 39 Austrian PCR ribotypes collected in the period 2006–2007 at 25 Austrian healthcare facilities. Capillary gel electrophoresis yielded up to 11 fragments per isolate and 47 ribotype patterns. All but one of the five PCR ribotypes of reference strains were clearly reflected in the chromatograms of capillary-based typing. Capillary gel electrophoresis divided 24 isolates belonging to PCR ribotype type 014 into seven subgroups, whereas subtyping the same isolates using multiple-locus variable-number tandem-repeat analysis yielded three unrelated subgroups, without obvious correlation to sr subgroups. Using a web-based software program (http://webribo.ages.at), we were able to correctly identify these 014 isolates by simply allocating the seven subgroup patterns to one ribotype, i.e. to PCR ribotype 014. We consider capillary gel electrophoresis-based PCR ribotyping to be a way of overcoming the problems associated with inter-laboratory comparisons of typing results, while at the same time substantially diminishing the hands-on time for PCR ribotyping.

## INTRODUCTION

*Clostridium difficile* is the most frequently identified cause of hospital-acquired diarrhoea and is the causative agent of pseudomembranous colitis. A new emerging hypervirulent strain of *C. difficile*, PCR ribotype 027, causes more severe disease and is associated with a higher mortality rate than other types (Kuijper *et al.*, 2006). This increased virulence is associated with two deletions in a toxin regulator gene and results in hyperproduction of toxins A and B. The incidence of *C. difficile*-associated disease due to type 027 is increasing in the USA, Canada, Asia and Europe (Kuijper *et al.*, 2006; Vonberg *et al.*, 2007; Pituch *et al.*, 2006; McDonald *et al.*, 2006). Other PCR ribotypes are also currently increasing, such as type 106 in the UK and type 078 in the Netherlands. Correct strain characterization is of the utmost importance for recognition of new circulating and emerging ribotypes. Several molecular subtyping methods have been investigated for strain characterization of *C. difficile*, including multilocus sequencing (Lemee *et al.*, 2004), multiple-locus variable-number tandem-repeat analysis (MLVA) (van den Berg *et al.*, 2007), restriction endonuclease typing (Kuijper *et al.*, 1987), toxinotyping (Rupnik *et al.*, 2001), repetitive extragenic palindromic elements PCR typing and PFGE (Northey *et al.*, 2005). However, PCR ribotyping, first described by Stubbs *et al.* (1999) and further developed by Bidet *et al.* (2000), has evolved as the dominant typing method. At least 150 different PCR ribotypes have been recognized but only a few of them are known to be human enteropathogens. Agarose gel-based PCR ribotyping has evolved as the most widely used method in European reference laboratories but is hampered by the lack of standardized type strain collections, which has led to the development of differing nomenclatures (Kato *et al.*, 2001; Spigaglia & Mastrantonio, 2004; Aspevall *et al.*, 2006). Since standardization and inter-laboratory exchange of data are difficult to achieve with conventional gel electrophoresis, we developed a *C. difficile* PCR ribotyping method based on capillary gel electrophoresis.

## METHODS

### Isolates.

A total of 141 well-characterized Austrian clinical isolates (Indra *et al.*, 2008) collected in the period 2006–2007 at 25 Austrian healthcare facilities were used in this study. Control strains for each of *C. difficile* ribotypes 001, 014, 017 and 027 were obtained from the Department of Medical Microbiology, University Medical Center, Leiden. The control strain for ribotype 053 was an Austrian isolate ribotyped by J. Brazier, Department of Microbiology, University of Cardiff, Wales, UK.

### Classic agarose gel-based ribotyping.

Primers 16S (5′-GTGCGGCTGGATCACCTCCT-3′) and 23S (5′-CCCTGCACCCTTAATAACTTGACC-3′) (VBC-Biotech) were used in classic agarose gel-based PCR ribotyping as described by Bidet *et al.* (1999). Briefly, DNA was extracted from cultures to a final volume of 50 μl using the MagNA Pure Compact (Roche) according to the manufacturer's instructions. Amplification reactions contained 25 μl HotStar Taq Master Mix (Qiagen), 1 μl (10 pmol μl^−1^) of each primer, 18 μl water and 5 μl DNA. Samples were amplified in a commercial PCR thermocycler running a 15 min 95 °C initial step for enzyme activation followed by 35 cycles of 1 min at 95 °C for denaturation, 1 min at 57 °C for annealing and 1 min at 72 °C for elongation, plus a 5 min 72 °C final elongation step. The amplified products were separated electrophoretically on 1.5 % agarose gels (Bio-Rad) for 4 h at 100 V. A 100–1000 bp ladder (Fermentas) was used as size standard every 10th lane.

Ribotype patterns different from those of the five reference strains available to the Austrian *C. difficile* reference laboratory (001, 014, 017, 027 and 053) were labelled previously with consecutive numbers and the prefix AI (Indra *et al.*, 2008). The strains included in this study belonged to ribotypes 001 (*n*=1), 014 (*n*=25), 017 (*n*=2), 027 (*n*=2), 053 (*n*=24), AI-1 (*n*=3), AI-2 (*n*=4), AI-3 (*n*=2), AI-5 (*n*=26), AI-6 (*n*=9), AI-8 (*n*=2), AI-9 (*n*=4), AI-10 (*n*=3), AI-14 (*n*=7), AI-17 (*n*=2), AI-19 (*n*=2), AI-23 (*n*=5), AI-50 (*n*=2) and one each of AI-4, AI-9-1, AI-10-1, AI-12, AI-15, AI-16, AI-18, AI-20, AI-21, AI-22, AI-24, AI-25, AI-26, AI-27, AI-29, AI-30, AI-31, AI-35, AI-48, AI-51 and AI-52.

PCR ribotyping pseudobands were analysed by eluting bands from agarose gels using the MinElute Gel Extraction kit (Qiagen) according to the manufacturer's instructions.

### Toxin typing.

Toxins were typed as described by van den Berg *et al.* (2004). Briefly, for detection of toxin A, region *tcdA* was tested using primers NKV011 (5′-TTTTGATCCTATAGAATCTAACTTAGTAAC-3′) and NK9 (5′-CCACCAGCTGCAGCCATA-3′). Toxin A-positive strains show a 2535 bp amplicon; toxin A-negative strains show deletions of 1700–1800 bp. For the detection of toxin B, region *tcdB* was tested using primers NK104 (5′-GTGTAGCAATGAAAGTCCAAGTTTACGC-3′) and NK105 (5′-CACTTAGCTCTTTGATTGCTGCACCT-3′) as described previously (Kato *et al.*, 1991, 1999); amplicons with a size of 204 bp were considered *tcdB*-positive. Amplification products were separated on a 1.5 % agarose gel (Bio-Rad) for 60–90 min at 100 V.

Presence of binary toxin was tested by detection of the binary toxin genes *ctdA* and *cdtB* using primers and conditions as described by Stubbs *et al.* (2000): for *ctdA*, forward primer ctdAfw (5′-TGAACCTGGAAAAGGTGATG-3′) and reverse primer ctdArev (5′-AGGATTATTTACTGGACCATTTG-3′); for *ctdB*, forward primer ctdBfw (5′-CTTAATGCAAGTAAATACTGAG-3′) and reverse primer ctdBrev (5′-AACGGATCTCTTGCTTCAGTC-3′). HotStar Master Mix (Qiagen) was used instead of standard *Taq* polymerase. Amplicons were detected on a 1.5 % agarose gel running for 60 min.

### Capillary gel electrophoresis-based ribotyping.

The same primers as listed above were used in capillary gel electrophoresis-based PCR ribotyping except that the 16S primer was labelled at the 5′ end with either carboxyfluorescein (FAM), hexachlorofluorescein (HEX) or tetrachlorofluorescein (TET). Prior to use, sample DNA was diluted 1 : 20 and 1.5 μl was used in reactions containing 25 μl HotStar Taq Master Mix, 0.3 μl (10 pmol μl^−1^) of each primer and 22.9 μl water. Samples were amplified in a commercial PCR thermocycler running a 15 min 95 °C initial enzyme activation step followed by 22 cycles of 1 min at 95 °C for denaturation, 1 min at 57 °C for annealing and 1 min at 72 °C for elongation, plus a 30 min 72 °C final elongation step. PCR fragments were analysed in an ABI 310 genetic analyser with a 41 cm capillary loaded with a POP4 gel (Applied Biosystems). Sample injection was at 5 kV over 5 s with a total running time of 30 min at 15 kV run voltage. A 50–625 bp TAMRA ladder (Chimerx) was used as an internal marker for each sample. The size of each peak was determined using Peak Scanner software 1.0 (Applied Biosystems). Ribotype patterns generated by capillary gel electrophoresis were named using the prefix 'sr'. A second master mix, AmpliTaq Gold (Applied Biosystems), was used for tests of reproducibility.

### MLVA typing.

The method described by van den Berg *et al.* (2007) was used for MLVA. Briefly, loci A6, B7, C6, E7, F3, G8 and H9 were amplified using fluorescence-labelled primers. The repeats were amplified using a single PCR protocol. DNA samples (1 μl) were amplified in a final volume of 20 μl containing 10 μl HotStart AmpliTaq Gold Master Mix and 1 μM of each primer. An initial enzyme activation step of 5 min at 95 °C was followed by 35 cycles of 30 s at 94 °C for denaturation, 30 s at 50 °C for annealing and 30 s at 72 °C for elongation, plus a final elongation step for 30 min at 68 °C. The forward primers were labelled at the 5′ end with FAM, HEX or TET. PCR fragments were analysed using multicoloured capillary gel electrophoresis on an ABI310 genetic analyser with a TAMRA500 ladder as an internal marker for each sample. The size of each peak was determined using Peak Scanner software version 1.0. BioNumerics software version 5.0 (Applied Maths) was used for cluster analysis by applying the unweighted pair group method with arithmetic mean (UPGMA) categorical analysis.

### Data analysis.

Data on PCR ribotype band sizes and MLVA were analysed using BioNumerics software version 5.0. The repeat numbers were analysed using BioNumerics software version 3.5 (Applied Maths) and UPGMA with arithmetic averages with the multistate categorical similarity coefficient. All markers were given an equal weight, irrespective of the number of repeats. Strains were considered to be related when they had an equal number of repeats in five out of seven markers. Fragment size was calculated using BioNumerics software for agarose-based electrophoresis and Peak Scanner software for capillary gel electrophoresis. Results from capillary gel electrophoresis-based PCR ribotyping were imported into BioNumerics 5.0. Peaks were counted as bands when they showed at least 10 % of the height of the highest peak of the individual run. Double peaks were counted only if they were separated by more than 1.5 bp. BioNumerics 5.0 software was used to apply an UPGMA Pearson correlation for cluster analysis.

### Web database.

A web-based database (http://webribo.ages.at) was created for capillary gel electrophoresis-based PCR ribotyping results. An error margin of ±4 bp was incorporated in the analysis algorithm of the database. With a web-based application, all users are able to enter their own data and receive a ribotype identification for each submitted isolate. Any unknown PCR ribotype pattern will be accepted if the isolate's chromatogram is submitted to the database administrator for proof of quality.

## RESULTS

**Fig. 1. f1:**
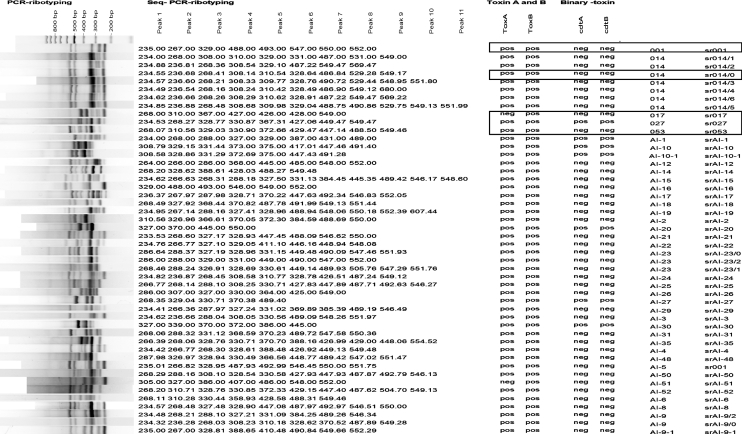
Ribotype patterns obtained by classic agarose gel-based electrophoresis (on the left), compared with those obtained using the capillary gel electrophoresis-based method (column labelled seq-PCR ribotyping). PCR ribotypes resulting from capillary gel electrophoresis received the prefix ‘sr’. Control reference strains are boxed.

**Fig. 2. f2:**
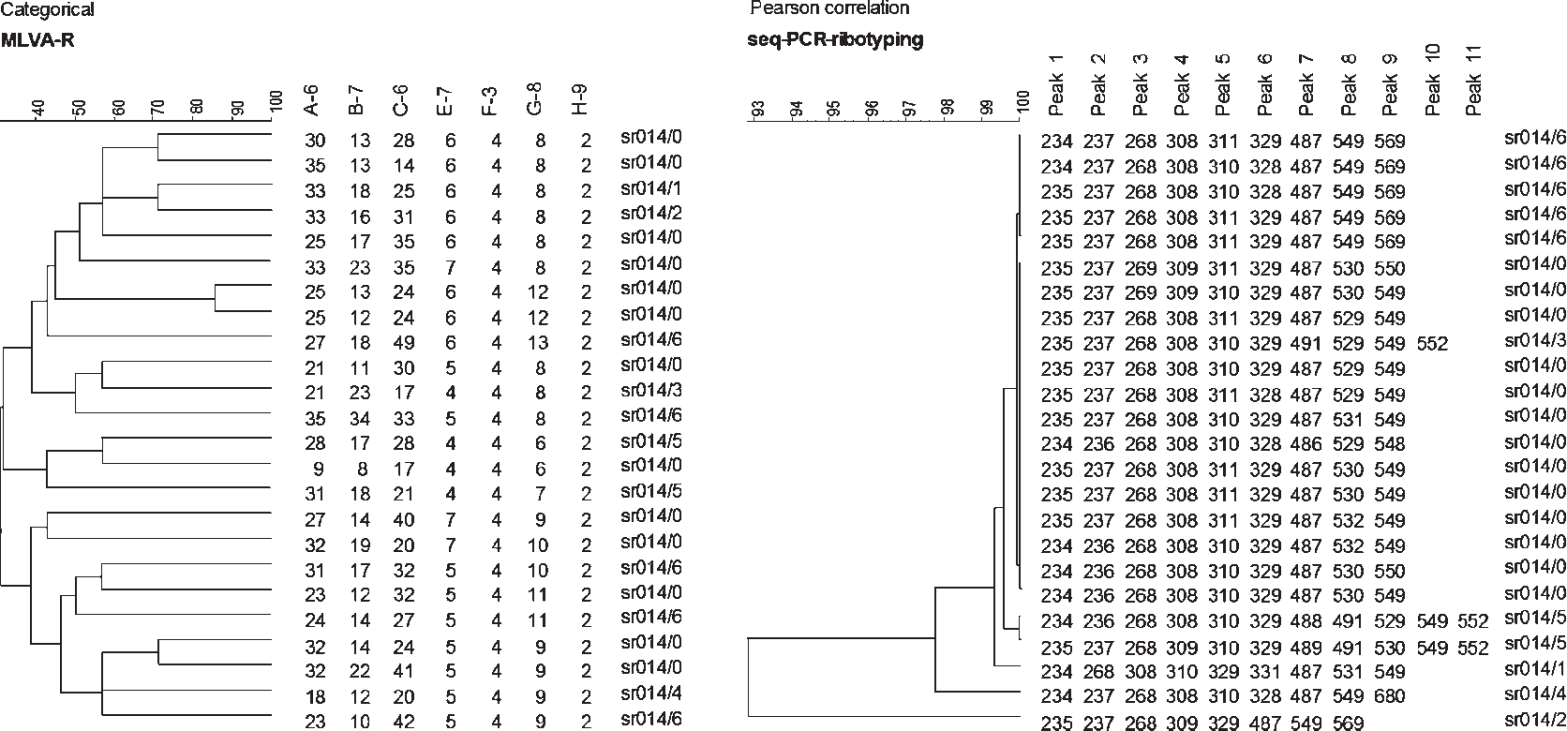
MLVA and capillary gel electrophoresis-based PCR ribotyping of 24 isolates belonging to ribotype 014. Capillary gel electrophoresis-based ribotyping yielded seven different 014 subgroups with assigned names sr014/0–sr014/6 (right), whereas MLVA subtyping of the same isolates yielded three unrelated subgroups without obvious correlation to sr subgroups (left).

Capillary gel electrophoresis yielded up to 11 fragments per isolate. Fragments had a minimum size of 233 bp and a maximum of 680 bp. Among isolates of one ribotype, the differences in minimal and maximal fragment size on conventional agarose-based gel electrophoresis were as high as 27 bp for certain fragments. Capillary gel electrophoresis results for individual peaks within one ribotype did not exceed deviations of more than a single base pair. Fig. 1 compares the 47 ribotypes resulting from capillary gel electrophoresis-based PCR ribotyping with the 39 ribotypes generated by classic agarose gel electrophoresis-based ribotyping. All but one of the five PCR ribotypes from reference strains were clearly reflected in the chromatograms of capillary-based typing. Capillary gel electrophoresis-based PCR ribotyping divided ribotype 014 (*n*=24) into seven distinct subgroups. Using a similarity index of 72 %, MLVA was capable of differentiating at least 20 subtypes (Fig. 2).

To test reproducibility, one isolate each of srAI-9, srAI-23/0, srAI-23/1 and srAI-23/2 was subcultured 10 times and DNA from the first and 10th passages was used in capillary gel electrophoresis-based PCR ribotyping. All isolates showed a stable number of fragments between the first and the last passage. The maximal difference in fragment size between the first and the 10th subculture was 0.98 bp (fragment 4 from AI-23/0) and the minimal difference was 0 bp. To test reproducibility with different master mixes, each subgroup of PCR ribotype 014 was tested in parallel using three different 16S primers (labelled with FAM, TET and HEX, respectively) and two different master mixes (HotStar and AmpliTaq Gold). Results of capillary gel electrophoresis-based PCR ribotyping were highly reproducible, independent of the brand of reagent used. The standard deviation per peak was ±0.5 bp with a median upper confidence interval of 0.83.

For all 26 isolates of ribotype AI-5, an agarose gel band 360–390 bp in size was not reflected in the chromatograms of capillary gel electrophoresis. Three AI-5 isolates were amplified with a fluorescence-labelled forward primer. The 360–390 bp fragment was eluted from agarose, and purified DNA was submitted to capillary gel electrophoresis separation. Again, the 360–390 bp band could not be found, but instead the method yielded three fragments 328, 234 and 266 bp in size. Fragments of these three sizes were found with both methods (classic and capillary-based typing) in all 26 AI-5 isolates and in the 001 isolate.

Capillary gel electrophoresis-based PCR ribotyping yielded the following 47 Austrian ribotype patterns for the 146 tested isolates: sr001 (*n*=28), sr014/0 (*n*=14), sr014/5 (*n*=2), sr014/6 (*n*=5), sr017 (*n*=2), sr027 (*n*=2), sr053 (*n*=23), srAI-1 (*n*=3), srAI-2 (*n*=4), srAI-3 (*n*=2), srAI-6 (*n*=9), srAI-8 (*n*=2), srAI-9-1 (*n*=2), srAI-10 (*n*=3), srAI-14 (*n*=7), srAI-17 (*n*=2), srAI-19 (*n*=2), srAI-23/2 (*n*=3), srAI-50 (*n*=3) and one each of sr014/1, sr014/2, sr014/3, sr014/4, srAI-10-1, srAI-12, srAI-15, srAI-16, srAI-18, srAI-20, srAI-21, srAI-22, srAI-23/0, srAI-23/1, srAI-24, srAI-25, srAI-26, srAI-27, srAI-29, srAI-30, srAI-31, srAI-35, srAI-4, srAI-48, srAI-51, srAI-52, srAI-9/0 and srAI-9/2. In Austria, the three ribotypes most frequently found are sr001, sr014 and sr053, which together account for approximately 50 % of all isolates typed (Indra *et al.*, 2008).

For all but two isolates, *in vitro* production of enterotoxin A and cytotoxin B was detected: one isolate of ribotype sr017 and one of srAI-51 were negative for enterotoxin A. Genes encoding binary toxin were found in 10 of the 141 isolates (7 %): in sr027 once, in srAI-1 three times, in srAI-10 three times, in srAI-10-1 once, in srAI-27 once and in srAI-30 once.

## DISCUSSION

PCR ribotyping patterns are based on size variations in the 16S–23S intergenic spacer regions of the bacterial rRNA (*rrn*) operon. According to Sadeghifard *et al.* (2006), the size of these regions ranges from 238 bp to 566 bp, which compares well with the fragment sizes found in our study (233–680 bp). Although the absence of an international type strain collection has led to varying nomenclature schemes, PCR ribotyping has evolved as the standard typing method for *C. difficile* in Europe (Kato *et al.*, 2001; Aspevall *et al.*, 2006) and has proven to be a valuable tool for epidemiological investigations of *C. difficile* outbreaks. Certain PCR ribotypes, however, can be divided into several subtypes using other molecular typing methods.

We developed a capillary gel electrophoresis-based subtyping assay that significantly reduces the hands-on time required for *C. difficile* PCR ribotyping. The results were highly reproducible, independent of reagent batches or brands used. Of the five PCR ribotypes from reference strains tested, all ribotypes but one were clearly reflected in the results of capillary-based typing.

Ribotype patterns generated by capillary gel electrophoresis were named using the prefix 'sr'. The patterns srAI-9-1, srAI-9/0 and srAI-9/2 represent unique strains, clearly distinguishable from each other, despite having similar agarose gel patterns (which explains the former type names AI-9 and AI-9-1) (Indra *et al.*, 2008). Capillary electrophoresis typing revealed that the former type AI-9 is formed from two independent sr types (AI-9/0 and AI-9/2) (Indra *et al.*, 2008). The binary toxin-positive isolates AI-10 and AI-10-1, showing very similar agarose gel patterns, yielded fragments of different sizes when tested by capillary gel electrophoresis, the names srAI-10 and srAI-10-1 reflecting the similarity revealed by the two methods.

Capillary gel electrophoresis divided PCR ribotype 014 into seven subgroups, a finding that may reflect either the existence of previously unrecognized new ribotypes or simply overdiscrimination. MLVA results did not support the existence of these seven sr subgroups. PCR ribotype 014 has been reported to be frequently recovered in France (Barbut *et al.*, 2007). Using a web-based software program, we were able to correctly identify 014 by simply allocating the seven subgroup patterns to one ribotype; that is, to PCR ribotype 014. This database (http://webribo.ages.at) also allows smooth data comparison between laboratories, as the *spa*-typing database (http://spaserver.ridom.de) does for *Staphylococcus aureus.*

Capillary gel electrophoresis typing showed that the Austrian PCR ribotype AI-5 is identical to PCR ribotype 001, despite a one-band difference on classic agarose gel electrophoresis. All AI-5 isolates tested in this study (*n*=27) had an identical pattern. Unfortunately, we did not have access to reference strains of the seven PFGE subtypes of PCR ribotype 001 described by Gal *et al.* (2005) and have no knowledge of their subtypeability using capillary gel electrophoresis-based ribotyping. The organization of the *rrn* operon might explain the extra band formation in the 27 isolates of AI-5 found in this study. Whereas in agarose gel-based electrophoresis double-stranded DNA fragments are separated, capillary gel electrophoresis separates denatured single-stranded DNA, stabilized with formamide. Sadeghifard *et al.* (2006) showed that the 16S–23S intergenic spacer region has a mosaic nature, like the whole genome of *C. difficile* itself (Sebaihia *et al.*, 2006). Following extraction of this extra band from the agarose gel and capillary gel electrophoresis, three fragments of different size were identifiable on the chromatogram. In our opinion, the band of the Austrian ribotype AI-5 is a product of incorrect hybridization of different 16S–23S intergenic spacer regions of similar sequence. By analysing the sequences of 55 different 16S–23S intergenic spacer regions, Sadeghifard *et al.* (2006) have shown that isolates of the same ribotype can have different intergenic spacer region sequences.

Capillary gel electrophoresis also revealed that two designated Austrian ribotypes, AI-9 and AI-23, were not unique ribotypes per se but each comprised various discrete ribotypes. In hindsight, both must be considered incorrect subtyping results of classic agarose gel electrophoresis. Such findings emphasize the importance of developing a European reference strain collection, as a prerequisite for establishing a common nomenclature.

Conventional agarose gel-based PCR ribotyping is easy to use and relatively cheap, but analysis of fragment lengths is hampered by poor resolution. By using 5′ fluorescence-labelled forward primers for amplification of the spacer regions, we were able to establish PCR ribotyping as a capillary gel electrophoresis-based assay. The new capillary-based assay allows inter-laboratory exchange of data without the need for cumbersome standardization of equipment, reagents and operating procedures. For institutes equipped with a capillary sequencer, the method reduces the cost of PCR ribotyping by drastically diminishing the hands-on time to about one-third of that required for the conventional agarose gel-based method.

At present, PCR ribotyping is the preferred typing method for *C. difficile* in Europe. Capillary gel electrophoresis-based PCR ribotyping might be a way of overcoming the problems associated with inter-laboratory comparisons of typing results. Furthermore, this new method could substantially improve a laboratory's capacity for *C. difficile* PCR ribotyping. Further studies with internationally accepted reference strains should endorse the value of capillary electrophoresis.

## Abbreviations

- FAM, carboxyfluorescein
- HEX, hexachlorofluorescein
- MLVA, multiple-locus variable-number tandem-repeat analysis
- TET, tetrachlorofluorescein
